# Disparities in oesophageal cancer risk by age, sex, and nativity in Kuwait:1980–2019

**DOI:** 10.1186/s12885-023-10770-0

**Published:** 2023-03-31

**Authors:** Saeed Akhtar, Ahmad Al-Shammari, Mohammad Al-Huraiti, Fouzan Al-Anjery, Salman Al-Sabah, Anjum Memon, Iqbal Siddique

**Affiliations:** 1 Department of Community Medicine and Behavioural Sciences, College of Medicine, Kuwait University, PO Box 24923, Safat, 13110 Kuwait; 2 Department of Surgery, Faculty of Medicine, McGill University, Montreal, QC Canada; 3 Ministry of Health, Kuwait City, Kuwait; 4 Department of Surgery, College of Medicine, Kuwait University, Kuwait City, Kuwait; 5 Department of Primary Care and Public Health, Brighton and Sussex Medical School, Brighton, UK; 6 Department of Medicine, College of Medicine, Kuwait University, Kuwait City, Kuwait

**Keywords:** Oesophageal carcinoma, Age-standardized incidence rate, Zero-inflated negative-binomial model, Incidence rate ratio, Kuwait

## Abstract

**Background:**

This cross-sectional cohort study assessed the inequalities in oesophageal carcinoma risk by age, sex and nativity in Kuwait: 1980–2019.

**Methods:**

Using oesophageal cancer incidence data from the Kuwait National Cancer Registry, relevant Kuwaiti population data and World Standard Population as a reference, age-standardized incidence rates (ASIR) (per 100,000 person-years) overall and by subcohorts were computed. The incident oesophageal cancer cases count was overdispersed with excessive structural zeros, therefore, it was analyzed using multivariable zero-inflated negative binomial (ZINB) model.

**Results:**

Overall ASIR of oesophageal cancer was 10.51 (95% CI:  6.62-14.41). The multivariable ZINB model showed that compared with the younger age category (< 30 years), the individuals in higher age groups showed a significant (*p* < 0.001) increasing tendency to develop the oesophageal cancer.  Furthermore, compared with the non-Kuwaiti residents, the Kuwaiti nationals were significantly (*p* < 0.001) more likely to develop oesophageal cancer during the study period. Moreover, compared with 1980-84 period, ASIRs steadily and significantly  (*p* < 0.005) declined in subsequent periods till 2015-19.

**Conclusions:**

A high incidence of oesophageal cancer was recorded in Kuwait, which consistently declined from 1980 to 2019. Older adults (aged ≥ 60 years) and, Kuwaiti nationals were at high risk of oesophageal cancer. Focused educational intervention may minimize oesophageal cancer incidence in high-risk groups in this and other similar settings. Future studies may contemplate to evaluate such an intervention.

## Introduction

Oesophageal cancer remains an important public health concern globally owing to its aggressive nature with an overall 5-year relative survival rate around 20% [1]. Worldwide, oesophageal cancer is the sixth most common malignancy accounting for over 500,000 cancer deaths each year [2, 3]. Oesophageal squamous-cell carcinoma (OSCC) and oesophageal adenocarcinoma (OA) are the two main histologic types of oesophageal cancer. OSCC is the predominant histological type of oesophageal cancer worldwide and is more common in Asia, whereas OA is main histologic type in high income western countries [4–6]. The known risk factors for oesophageal cancer are tobacco smoking, excessive alcohol consumption, low fruit and vegetable intake, high intake of red meat, the consumption of very hot beverages, genetic factors, gastro-oesophageal reflux, and obesity [7, 8]. Over the past few decades, linear declines in oesophageal cancer incidence have been recorded worldwide [9–11], whereas, in some regions, its incidence has been stable [2, 9]. However, the recent global trends have not been assessed in many regions including the middle eastern countries specifically in Kuwait. Therefore, this cross-sectional cohort study assessed the secular trends in population-level oesophageal cancer risk by age, sex, and nativity (1980–2019) in Kuwait.

## Methods

In this cross-sectional cohort study, oesophageal cancer incidence data were obtained either from the Kuwait Cancer Control Center Registry (1980–2016) or projected (2017 to 2019). For each oesophageal cancer patient, we obtained the date of birth, age (years) at diagnosis, sex (male, female) and nativity (Kuwaiti, non-Kuwaiti). Relevant Kuwaiti population data were obtained from Public Authority for Civil Information, Ministry of Interior, Kuwait. Mid-year population counts for each year and by subcohorts were defined as the person-years. Age-standardized incidence rates (ASIR) (per 100,000 person-years) of oesophageal cancer overall, by year for unstratified data and by the subcohorts defined by cross-classification of period of diagnosis (5-year groups), age at diagnosis (10-years age brackets), sex (male or female) and nativity (Kuwaiti or non-Kuwaiti) were computed using World Standard Population as a reference ([12]. The trends in ASIR (per 100,000 person-years) across age, sex, nativity and periods were evaluated.  Outcome variable i.e., count of incident oesophageal cancer cases was overdispersed with excessive structural zeros, therefore, it was analyzed using zero-inflated negative binomial (ZINB) model. Univariable and multivariable ZINB models were fitted to the data and ZINB models’ coefficients and their corresponding standard errors were used to compute both unadjusted and adjusted incidence rate ratios (IRR) and their 95% confidence intervals (CI) respectively, which were used for the models’ interpretation.

## Results

**Fig. 1:**
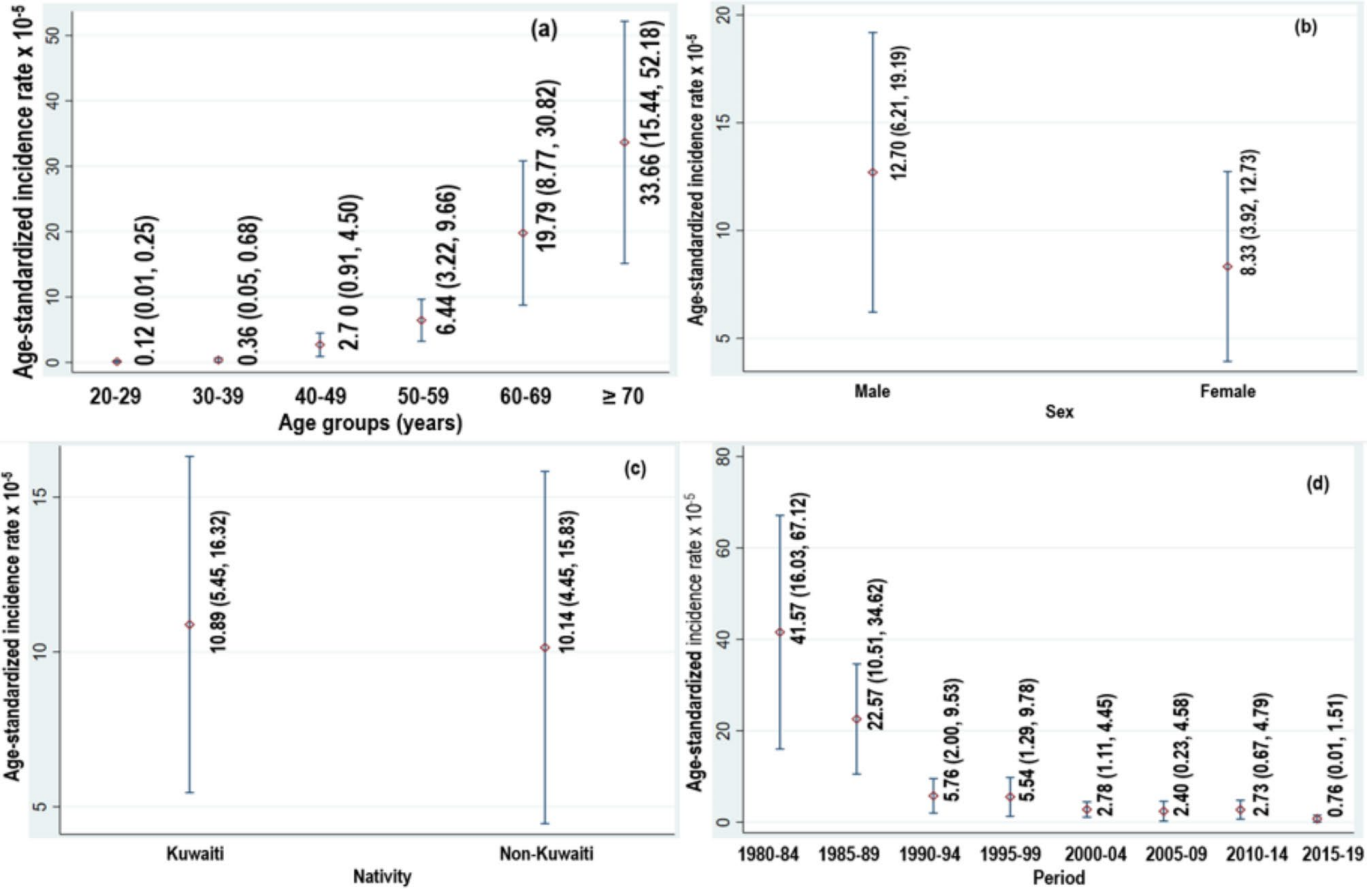
Mean age-standardized incidence rates (ASIRs) (per 100,000 person-years) (95% confidence intervals) of oesophageal carcinoma by age, sex, nativity and period in Kuwait:1980–2019. (Vertical bars represent mean ASIRs and corresponding 95% confidence intervals for ASIRs).

**Fig. 2:**
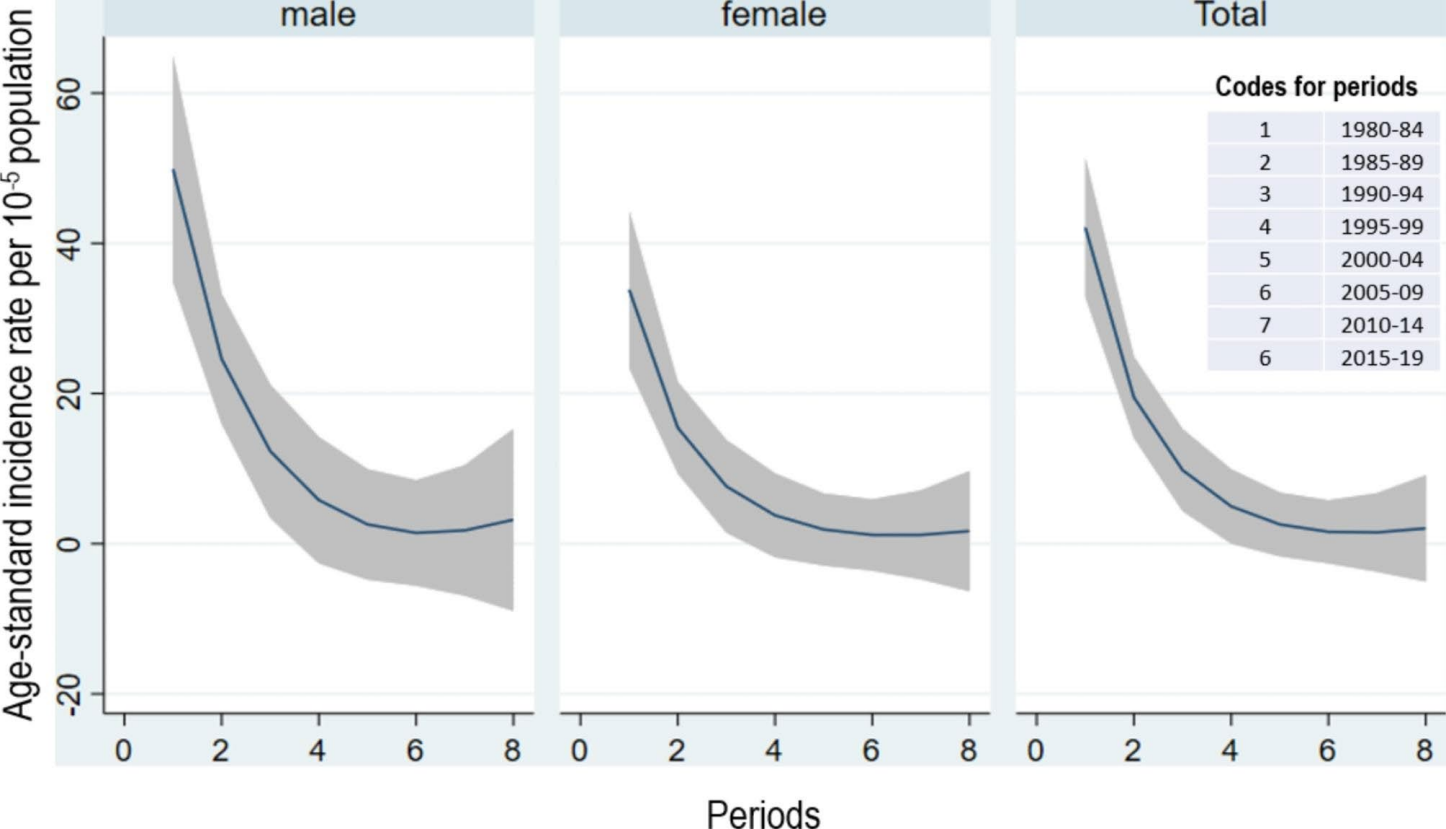
Declining trends of age-standardized incidence rates of oesophageal cancer by sex and total population in Kuwait: 1980–2019

During the study period, a total of 496 cases of oesophageal cancer in 12.8 million person-years at risk were diagnosed. Of these, 269 (54.23%) were OSCC, 147 (29.64%) AC and 80 (16.13%) cases were histopathological unspecified. Subsequently, all the histopathological types were grouped as oesophageal cancer cases. The overall ASIR (per 100,000 person-years) of oesophageal cancer during the study period was 10.51 (95% CI:  6.62-14.41). The ASIRs (per 100,000 person-years) by age, sex, nativity and time periods are shown in Fig. 1. The highest ASIR (per 100,000) of oesophageal cancer was among 70 years old or older individuals (ASIR = 33.66; 95% CI: 15.44, 52.18). The ASIR (per 100,000 person-years) of oesophageal cancer was higher among males (ASIR = 12.70; 95% CI: 6.21, 19.19) than females (ASIR = 8.33 ; 95% CI: 3.92, 12.73). The highest ASIR (per 100,000 person-years) of oesophageal cancer was observed during 5-year period of 1980-84 followed by a consistently declining trend in subsequent 5-year periods. This declining trend was consistent across males and females as well (Fig. 2).

The multivariable ZINB model revealed that compared with the individuals younger than 30 years, the oesophageal cancer risk significantly (*p* < 0.001) increased through the higher age groups (Table 1). The oesophageal cancer risk among males compared with females was only marginally significant (adjusted IRR = 1.22; 95% CI:  0.99, 1.49; *p* = 0.058). Furthermore, compared with the non-Kuwaiti residents, the Kuwaiti nationals were significantly at higher risk of developing oesophageal cancer during the study period (adjusted IRR = 1.83; 95% CI: 1.49, 2.24; *p* < 0.001). Moreover, compared with 1980-84 period, in each of the subsequent 5-year periods, adjusted IRRs showed a significant (*p* < 0.005) and nearly a consistent declining tendency (Table 1).

**Table 1:** Mutlivariable zero-inflated negative binomial model of esophageal cancer risk by age, sex, nativity and period in Kuwait:1980–2019

Characteristic	Unadjusted IRR *^,a^	95% CI**	Adjusted IRR	95% CI	*p*-value
Age (years) at diagnosis					
< 30	1.00	–	1.00	–	< 0.001
30–39	2.81	0.94–8.35	2.11	1.06–4.22	< 0.001
40–49	23.80	8.51–66.56	10.01	5.37–18.64	< 0.001
50–59	69.45	25.62–188.62	33.89	18.49–62.09	< 0.001
60–69	220.92	82.02–595.02	92.40	50.42–169.34	< 0.001
≥ 70	382.55	141.92–1031.18	262.52	143.52 – 480.20	< 0.001
Sex					
Female	1.00	–	1.00	–	
Male	1.51	0.77–2.94	1.22	0.99–1.49	0.058
Nativity					
Non-Kuwaiti	1.00	–	1.00	–	< 0.001
Kuwaiti	1.16	0.60–2.27	1.83	1.49–2.24	
Period					
1980-84	1.00	–	1.00	–	–
1985-89	0.54	0.18–1.56	0.60	0.42–0.85	0.004
1990-94	0.13	0.05 – 0.39	0.11	0.08–0.15	< 0.001
1995-99	0.13	0.05 – 0.39	0.08	0.06–0.12	< 0.001
2000-04	0.06	0.02 – 0.18	0.05	0.04–0.08	< 0.001
2005-09	0.05	0.02 – 0.16	0.04	0.03–0.06	< 0.001
2010-14	0.06	0.02 – 0.19	0.04	0.03–0.06	< 0.001
2015-19	0.01	0.00–0.03	0.01	0.01–0.02	< 0.001

*Incidence rate ratio, **CI: confidence interval; ^a^ IRR for study periods exhibited significant exponenatial increase

(Anderson-Darling goodness-of-fit statistic = 0.721; *p* = 0.204)

## Discussion

The results showed that during the past four decades, the overall ASIR (per 100,000 person-years) of oesophageal cancer was 10.51 in Kuwait. This estimate is comparable with those recently reported in Eastern Asia (11.1), but is nearly twice as much of the estimates reported from South-Central Asia (4.8), sub-Saharan Africa (4.2) and other world regions [13]. This global variation in ASIRs of oesophageal cancer may be owing to varying lifestyle and dietary patterns. The two established risk factors for oesophageal cancer are excessive alcohol consumption and tobacco smoking. Parenthetically Kuwait ranks very high worldwide for tobacco consumption prevalence in men (33.7%) and women (4.7%) [14], with parallel high ASIRs of oesophageal cancer, which indeed fulfils the Bradford Hill’s criterion of ‘coherence’ for causal link between tobacco smoking and oesophageal cancer.

The multivariable ZINB model showed that increasing age was associated with the increasing trend in the oesophageal cancer risk, which reflects a cumulative effect of carcinogenic exposures. The modification of known risk factors for oesophageal cancer may lessen the risk later in the life. Moreover, males had marginally increased oesophageal cancer risk than females, which outwardly related to sex hormones and lifestyle factors including smoking and dietary factors [7, 8]. Additionally, over the past four decades, oesophageal cancer risk consistently declined in Kuwait. Towards the end of twentieth century oesophageal cancer risk had been increasing in both sexes in USA, Europe, Japan and China [15, 16], which in recent decades showed a declining trend in these regions as well [15]. Similarly, downward trends with varying magnitudes in oesophageal cancer incidence were recorded in Hong Kong [17], and China [18]. These declining trends in oesophageal cancer incidence ostensibly is due to varying distributions of contributory risk factors across different populations [15, 19]. For example, in Kuwait, tobacco smoking by any mode including cigarettes, cigars and pipes showed a consistently declining trend with varying rates (5–6%) from 2000 to 2018 and stabilized thereafter, which seems to concur with the decreasing trend of oesophageal cancer in Kuwait [20].

In conclusion, an overall high oesophageal carcinoma ASIR was recorded. ASIRs consistently declined from1980 to 2019. Older adults (≥ 60 years) and Kuwaiti nationals were high-risk groups for oesophageal cancer. An educational intervention based on the known risk factors may alleviate oesophageal carcinoma risk in this and similar settings. Future studies may contemplate to evaluate the effect of such an intervention.

## Data Availability

The dataset used in this study can be made available on a reasonable request to the corresponding author (SA) and additional approval of the Director, Kuwait Cancer Control Center Registry.

## Declarations

### Competing of interests

None declared.
